# Effect of Land‐Use on Hantavirus Infection Among Introduced and Endemic Small Mammals of Madagascar

**DOI:** 10.1002/ece3.70914

**Published:** 2025-04-07

**Authors:** Jérémy Dubrulle, Kayla Kauffman, Voahangy Soarimalala, Toky Randriamoria, Steven M. Goodman, James Herrera, Charles Nunn, Pablo Tortosa

**Affiliations:** ^1^ Unité Mixte de Recherche Processus Infectieux en Milieu Insulaire Tropical (UMR PIMIT) Université de la Réunion, CNRS 9192, INSERM 1187, IRD 249 Sainte‐Clotilde Réunion Island France; ^2^ Department of Evolutionary Anthropology Duke University Durham North Carolina USA; ^3^ Department of Ecology, Evolution, and Marine Biology University of California Santa Barbara California USA; ^4^ Association Vahatra Antananarivo Madagascar; ^5^ Field Museum of Natural History Chicago Illinois USA; ^6^ Duke Global Health Institute, Duke University Durham North Carolina USA

**Keywords:** disease ecology, land‐use, Madagascar, *Orthohantavirus*, *Rattus rattus*, viral ecology

## Abstract

Hantaviruses are globally distributed zoonotic pathogens capable of causing fatal disease in humans. Addressing the risk of hantavirus spillover from animal reservoirs to humans requires identifying the local reservoirs (usually rodents and other small mammals) and the predictors of infection, such as habitat characteristics and human exposure. We screened a collection of 1663 terrestrial small mammals and 227 bats for hantavirus RNA, comprised of native and non‐native species from northeastern Madagascar, trapped over 5 successive years. We specifically investigated the influence of diverse habitat types: villages, agricultural fields, regrowth areas, secondary and semi‐intact forests on infection with hantaviruses. We detected Hantavirus RNA closely related to the previously described Anjozorobe virus in 9.5% of *Rattus rattus* sampled, with an absence of detection in other species. Land‐use had a complex impact on hantavirus infections: intensive land‐use positively correlated with the abundance of *R. rattus* and the average *R. rattus* body size varied between habitats. Larger individuals had a higher probability of infection, regardless of sex. Thus, villages and pristine forests which host the smallest, and hence, least infected rats, represent the lowest risk for hantavirus exposure to people while flooded rice fields which were home to the largest rats, and subsequently most infected rats, represent the greatest exposure risk. These findings provide new insights into the relationship between rat ecology and the gradients of hantavirus exposure risk for farmers in northeastern Madagascar as they work in different land‐use types.

## Graphical abstract

Addressing the risk of Hantavirus spillover from animal reservoirs to humans requires identifying the local reservoirs and the predictors of infection. We screened a collection of 1880 small mammals for Hantavirus RNA from northeastern Madagascar, and specifically investigated the influence of diverse natural or anthropized habitats as well as animal morphometrics on infection prevalence. Rats were the only detected reservoirs and their mass positively correlated with infection prevalence. Average *R. rattus* body size varied between habitats, directly impacting infection prevalence. Conversely, infection prevalence was highest in agricultural areas and minimal in secondary forests.

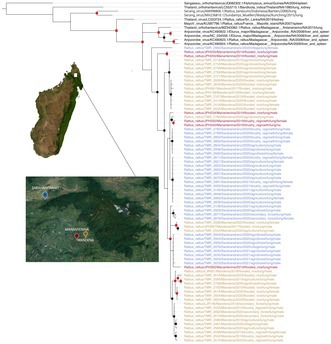

## 1 Introduction

Zoonotic diseases represent a major threat to human health, with hundreds of thousands of deaths and millions of infections occurring annually (Cascio et al. 2011). Of the many zoonoses that impact humans, hantaviruses are a growing threat due to their increasing global incidence and high mortality rate (Johnson et al. 2013). Particularly, the Old World hantaviruses are responsible for more than 100,000 infections per year (Avšič‐Županc, Saksida, and Korva 2019), and disproportionately affect low‐income, rural areas (Cascio et al. 2011). Of the Old World strains, Seoul *Orthohantavirus* has caused more than 1.6 million infections in China since the 1950's (Cascio et al. 2011).

Humans are primarily exposed to hantaviruses through aerosolized excrement of infected rodents, which is broadly associated with activities that bring humans into contact with these animals and where rodent densities are relatively high (Watson et al. 2014). In the Old World hantaviruses, the disease associated with infection in humans is hemorrhagic fever with renal syndrome and has a mortality rate of 15% (Schmaljohn and Hjelle 1997). Antiviral drugs and vaccines are not yet available, with control instead relying on exposure prevention (Brocato and Hooper 2019). Thus, understanding the enzootic ecology of these viruses, factors facilitating spillover into non‐reservoir hosts, as well as viral evolution, are important aspects to prevent human infections (Jonsson, Figueiredo, and Vapalahti 2010).

*Orthohantavirus* species from the Hantaviridae family form a diverse genus of RNA viruses with a vast set of enzootic reservoir hosts. Old World hantaviruses are primarily harbored by Muridae (rodents) and *Myodes* (voles; Jonsson, Figueiredo, and Vapalahti 2010). Other reservoirs include shrews (Soricidae) in Guinea (Klempa et al. 2007; Guo et al. 2013), China (Guo et al. 2013), Korea (Lee et al. 2017), Vietnam (Song et al. 2007), and Hungary (Kang, Bennett, Sumibcay, et al. 2009) and bats in China (Guo et al. 2013), Ivory Coast (Sumibcay et al. 2012), and Sierra Leone (Weiss et al. 2012). Phylogenetic studies reveal co‐divergence, reassortment, and host‐switching between members of the cricetid rodents, particularly *Lemmus sibirica* and *Microtus fortis* (Vapalahti et al. 1999) and between the Talpidae and Soricidae families (Kang, Bennett, Dizney, et al. 2009), all of which account for the high diversity and worldwide distribution of hantaviruses.

Climatic factors such as fluctuations in temperature and rainfall in combination with local human modifications in landscape structure affect reservoir abundance (reviewed in Prist, D'Andrea, and Metzger 2017), which in turn changes the dynamics of hantavirus infections and human exposure risk (Klempa 2009). Abundance of reservoir hosts and infection rates varies seasonally with food availability (Nsoesie et al. 2014; Prist, D'Andrea, and Metzger, 2017). Habitat availability (Suzán et al. 2008) and fragmentation (Ganzhorn 2003; Goodin et al. 2006; Fialho, Cerboncini, and Passamani 2019) also impact community composition, with fragmented areas and agricultural fields often having denser populations of introduced commensal species such as mice ( *Mus musculus* ) and rats (*Rattus norvegicus and* *Rattus rattus* ; Umetsu and Pardini 2007). Deforestation also benefits invasive generalist species such as introduced rodents, which in turn tends to decrease the diversity of endemic and native species in the local mammal communities (Pardini et al. 2010).

Madagascar provides a valuable context to investigate the effect of anthropogenic disturbance on hantavirus infection in small mammals as the island is experiencing rapid deforestation and habitat fragmentation (Vieilledent et al. 2018). Madagascar's exceptional biodiversity and levels of endemism are due to isolation in deep geological time (Antonelli et al. 2022). This landmass is home to a unique native small mammal fauna, including 46 species of bats (80% endemic), 31 species of endemic tenrecs (Geogalinae, Oryzorictinae, and Tenrecidae), and 28 species of endemic rodents (subfamily Nesomyinae). Non‐native Muridae rodents (*Rattus* and *Mus*) and Soricidae shrews (*Suncus*) also inhabit the island. This isolation and diversity may explain the evolution of unique hantavirus lineages described from the island such as Anjozorobe virus (ANJZV), a variant of Thailand virus (*Orthohantavirus thailandense*), which was found in introduced *Rattus rattus* and endemic *Eliurus majori* in Madagascar (Reynes et al. 2014; Raharinosy et al. 2018; Kikuchi et al. 2021). Human exposure to ANJZV in Madagascar is estimated at 2.7% nationally, with higher seroprevalence (7.2%) in villages adjacent to forests (Rabemananjara et al. 2020). A closely related hantavirus lineage, called *MayoV*, has also been described in *R. rattus* on the neighboring island of Mayotte (Filippone et al. 2016).

Here, we investigated the ecology of hantavirus infection in rural northeastern Madagascar in relation to mammalian community composition and land use patterns. We surveyed a wide range of terrestrial small mammals and bats for hantavirus over a 5‐year period along gradients of land‐use in and around Marojejy National Park in northeastern Madagascar. We focused on areas of mixed land‐use surrounding three villages, including swidden agriculture that results in the clearing of forests and a fragmented landscape. Remnant forests are largely restricted to protected areas, and secondary forests are embedded in a matrix of brushy regrowth and agricultural fields (e.g., rice fields, vanilla agroforestry). Previous work showed that these habitat modifications have resulted in higher abundances of commensal small mammals, specifically non‐native rodents and shrews around one of our study villages, as compared to the nearby protected forest (Herrera et al. 2020). These factors are anticipated to influence hantavirus ecology by modifying the abundance and diversity of mammal hosts and by increasing the risk of contact with hantavirus‐infected substrates and materials (reviewed in Tian and Stenseth 2019).

As found in other tropical settings, we hypothesized that hantavirus infections would be more common during the rainy season, driven by increased population densities of small mammal hosts at this time of year (Scobie et al. 2024). We also hypothesized that viral infection rates in small mammals would be higher in large, adult males, as observed in other studies, which link sexual selection behaviors—competing for resources and mates—to increased territory size and physical contacts, and thus, increased viral exposure and infection (Jonsson, Figueiredo, and Vapalahti 2010; King et al. 2014; Mori et al. 2017). We expected that the proportion of animals infected with hantavirus would increase with agricultural use, as a result of increased *R. rattus* abundance. Lastly, we used the variability in hantavirus infection across this suite of land‐use types to better understand why Raharinosy et al. (2018) found lower viral infection rates within houses than outside of them in Madagascar.

## 2 Material and Methods

### 2.1 Ethics Statement and Sample Collection

Lung tissue samples from wild small mammals were collected between 2017 and 2021 in the vicinity of three villages adjacent to Marojejy National Park in the SAVA Region of northeastern Madagascar, following a standard grid‐trapping protocol detailed below. The village of Mandena (14.477049° S, 49.8147° E) and its surroundings were sampled during the dry season (September–December) in 2017 and 2019, during the wet season (March–May) in 2020, and in the transitional dry‐wet season (June–August) in 2018 and 2020. A second village, Manantenina (14.497213° S, 49.821347° E), was sampled only in the transitional season (2019) while a third village, Sarahandrano (14.607567° S, 49.647759° E), was sampled during the dry (2020) and wet (2021) seasons. Bats were trapped along flyways located on or adjacent to the trap grids and similar habitat types using harp traps and mist nets. Bats were also captured from caves in the area using butterfly nets. Bat trapping occurred during September–November 2019 in Mandena (dry season) and March–May 2021 in Sarahandrano (wet season).

Small mammal traps were installed in different land‐use types, including semi‐intact forest within the national park, as well as secondary forest and agroforestry outside the park, and agricultural fields, flooded rice fields, and brushy regrowth (fallow areas after swidden cultivation) around villages. Traps were also placed in houses within villages adjacent to the trap grids. Sampling methods varied by location and trap grid; trap grids prior to September 2019 were 90 m^2^ and after that date were 100 m^2^ and consisted of metal (Sherman, model LFAm Tallahassee, Florida, USA) and mesh (Tomahawk, model 201, Hazelhurst, Wisconsin, USA) live traps placed 10 m apart. At each sampling site, two pitfall lines of 100 m in length, composed of 15 L buckets placed 10 m apart and the line bisected by a vertical plastic drift fence partially buried. The pitfall lines were located 20–50 m from the trap grid. Most grids were sampled for six consecutive nights, but where abundance was low, longer trapping periods were needed (up to 15 nights), and trapping within houses was limited to five nights. All non‐native animals and a subset of native animals were collected. Tissue samples were stored in 70% ethanol and placed in long‐term storage for 1–4 months at −20°C until the molecular analyses were conducted.

All animals were processed using the same methods and samples were stored in the same conditions until further laboratory analysis. All procedures were approved by IACUC at Duke University (protocol number A002‐17‐01, 2017–2019, A262‐19‐12 2019–2021) and by Malagasy authorities (No. 289/17, 146/18, 280/19, 57/20, 191/20, 307/21—MEEF/SG/DGF/DSAP/SCB).

### 2.2 Nucleic Acids Extraction

Lung samples were rehydrated overnight at 4°C in 1.5 mL of autoclaved milliQ water. Then 25–50 mg of rehydrated tissue was cut into small pieces with a sterile disposable scalpel blade and transferred into individual 2 mL Eppendorf tubes containing 180 μL of ATL and 20 μL of proteinase K provided by the IndiSpin QIAcube HT Pathogen Kit (Qiagen, Courtaboeuf, France). Tubes were incubated between 6 and 12 h at 56°C until full proteolysis was achieved. Extraction was then performed in 96 well plates using the Qiagen Cador pathogen kit and QiaCube XT robot following the manufacturer's instructions. Nucleic acids were collected in a 200 μL elution buffer and stored at −80°C.

### 2.3 Hantavirus Detection

Reverse transcription was conducted with 10 μL of eluted nucleic acids using the ProtoScript II Reverse Transcriptase (New England Biolabs, Massachusetts, USA). For each sample, RNA was denatured at 70°C for 5 min in a mix containing 1 μL of dNTP, 1 μL of Rnase free water, and 0.5 μL of random primers and thawed in a cold block. Then, a second mix was added, containing 4 μL of protoscript buffer, 2 μL of 10X DTT, 1 μL of the reverse transcription enzyme, and 0.5 μL of RNAsin (New England Biolabs, Massachusetts, USA), resulting in a total volume of 20 μL. The mix was incubated at 25°C for 10 min, 42°C for 50 min and 65°C for 20 min.

cDNAs were used as a template in a previously published nested‐PCR targeting the L‐segment coding for the RNA‐polymerase RNA‐dependent (Klempa et al. 2006). The mix contained 12.5 μL of GoTaq G2 Hot Start Polymerase (Promega, Wisconsin, USA), 1 μL of degenerated primers at 10 μM (HAN‐L‐F1 and HAN‐L‐R1 for primary PCR, HAN‐L‐F2 and HAN‐L‐R2 for secondary PCR). 2 μL of cDNA was used for the primary PCR's template. The secondary PCR was prepared using 0.5 μL of the primary PCR product as DNA template. Thermal cycling conditions were identical for both primary and secondary PCR and were as follows: nucleic acids were denatured at 95°C for 5 min followed by 2 cycles at 94°C for 45 s, 46°C for 45 s and 72°C for 60 s, 2 cycles at 94°C for 45 s, 44°C for 45 s, and 72°C for 60 s, then 30 cycles at 94°C for 45 s, 42°C for 45 s, and 72°C for 60 s before finalizing the PCR at 72°C for 10 min. DNA was visualized using a 1.8% TBE agarose gel stained with Gel Red (Biotium, Fremont, California, USA).

### 2.4 PCR Targeting Hantavirus in Endemic Hosts

Additional primers were developed to detect hantavirus RNA that might be hosted by endemic Malagasy small mammals. Primers were either referenced from the literature (Klempa et al. 2006; Table S1) or newly designed using available reference sequences of ThaiV (MZ343362.1), MayoV (KU587796.1), and ANJZV (LC553724.1, NC_034556.1, KC490924.1, KC490923.1, and KC490922.1). Three semi‐nested PCR schemes were used to screen endemic mammals and their associated nucleotide sequences are presented in Table S1. The lack of positive hantavirus detection in endemic mammals resulted in the need to test newly designed primers on positive *R. rattus* found in this study, through a nested PCR protocol. Primer pairs that did not successfully amplify these *R. rattus* hantaviruses were excluded from subsequent analyses, resulting in three semi‐nested PCR systems, which were all used following these PCR conditions: initial denaturation at 95°C for 5 min was followed by 35 cycles of 45 s at 95, 45 s annealing at 50°C and 1 min elongation at 72°C. PCR ended with a final elongation step of 10 min at 72°C. PCR products were visualized on a 2% agarose gel and gel bands with the expected size were gel purified (QIAquick Gel Extraction Kit) and used for Sanger sequencing (Genoscreen, Lille, France).

### 2.5 Phylogenetic Analyses

Positive nested PCR samples were Sanger sequenced on both DNA strands at Genoscreen (Lille, France). Chromatograms were manually edited using Geneious 9. Sequences of the partial L segment, 347 bp, are available on GenBank under accession numbers OP328829‐OP328903 (Table S5). We included in the analyses sequences from reference HantaV strains belonging to lineages directly related to ThaiV viruses, and using Sangassou virus as an outgroup. The final tree was based on a set of 85 sequences of 347 bp composed of 11 external reference sequences related to ThaiV hantavirus, and 74 sequences generated in the context of this study. We imported our dataset into Datamonkey Adaptive Evolution server to identify the presence of recombinant strains (https://www.datamonkey.org/). The most appropriate phylogenetic model was determined using T93 + G + I generated using MEGA11 (alignment in Supporting Information). Phylogenetic signal was checked for with DAMBEE and the tree was built on 20,000,000 iterations. MrBayes package on Geneious 9.1 was fed with the aforementioned settings while other settings were set to default values. Reference sequences were trimmed to 347 bp and all sequences were aligned using the E‐INS‐i algorithm part of the “MAFFT Alignment” plugin on Geneious 9 (Katoh and Standley 2013).

### 2.6 Statistical Analyses

All analyses were performed in R version 4.3.0 (R Core Team, 2024). All pairwise comparisons were made using a significant level of 0.05. Only *R. rattus* was considered in the analyses as hantaviruses were only detected in this species. We used generalized linear mixed‐effects models (GLMMs) with a binomial error structure to investigate the effects of environmental and individual variables on hantavirus infection in *R. rattus* . To examine the random effect of trap grid identity, a unique identifier given to each trap grid installation was included in all models to control for the non‐independence of animals captured in the same grid. We considered the fixed environmental effects of season (dry, wet, and transitional), village, direct trap distance to the village, and habitat type (semi‐intact forest, secondary forest, agroforestry, brushy regrowth, agriculture, flooded rice fields, and village). Individual‐level fixed effects considered were sex, age based on tooth eruption and wear (sub‐adult and adult), mass (g), head‐body length (mm), body condition score, and the interaction term between mass and head‐body length. The body condition was calculated using mass per length cubed (Fulton's index) and served as an indicator of an individual's health. A final fixed effect, the number of *R. rattus* captured during each trap grid installation (e.g., flooded rice field in Mandena during the dry season 2019), was also used as an approximation for animal density. To assess whether variation in hantavirus infection rates between habitat types could be explained by individual‐level traits, environmental traits, or rat density, we used a model containing the combinations of those traits (environmental + demographic, environmental + number of *R. rattus* , and environmental + demographic + number of *R. rattus* ). The data used in all models was subset to include observations containing all representative metadata. Furthermore, trapping effort and sampling methods in villages differed substantially from all other sites. Thus, these variables were excluded in the models that included animal density. All models were also rerun with the complete data set available for covariates included to verify the robustness of the results.

Global models were fitted using the glmmTMB package, version 1.1.7 (Brooks et al. 2017) and diagnostic tests were performed using the DHARMa package, version 0.4.6 (Hartig 2022). We used the MuMIn package, version 1.47.5 (Bartoń 2024) ‘dredge’ function to identify the best models based on the corrected Akaike information criterion (AICc), then retained all models in the 95% cumulative sum weight confidence set to find the cumulative sum AICc weights (importance) for each predictor and calculate model‐averaged parameter estimates. We used the full averaged model to perform Tukey‐adjusted post hoc pairwise comparisons using the emmeans package, version 1.8.6. The proportion of variance explained by the marginal (fixed) effects of best model (Nakagawa's marginal *R* ^2^) was found using the ‘r2_nakagawa’ function in the performance package (Lüdecke et al. 2021).

## 3 Results

### 3.1 Hantavirus Infections in Small Mammals

We trapped 1681 small mammals, of which 1663 were tested for hantavirus infection. The tested samples consisted of 72.9% (*N* = 1213) non‐native and 27.1% (*N* = 450) endemic mammals (Table S2). We also captured 350 bats, of which 64.9% (*N* = 227) were tested for hantavirus infection. Introduced *R. rattus* represented 47.8% (*N* = 794) of the tested animals and the only species in which hantavirus was detected. All endemic species of small mammals and bats tested negative through nested PCR.

To examine possible false negative PCRs resulting from infections with distinct hantaviruses, we tested most endemic mammals, as well as introduced shrews, with alternative PCR schemes. For this, we first validated alternative PCR schemes with known positive *R. rattus* from this study. Seven out of 16 primers produced amplicons of the expected size and were further tested on subsequent endemic rodents. Samples from 214 endemic mammals representing 12.8% of all trapped mammals including *Microgale brevicaudata* (*N* = 176), *Eliurus* spp. (*N* = 9), *Setifer setosus* (*N* = 29), *Suncus murinus* (*N* = 55), and native bats (*N* = 141, see Table S2) were tested. None of these samples tested positive using these alternative end‐point PCR schemes.

### 3.2 Genetic Diversity of the Detected Hantaviruses

**FIGURE 1:**
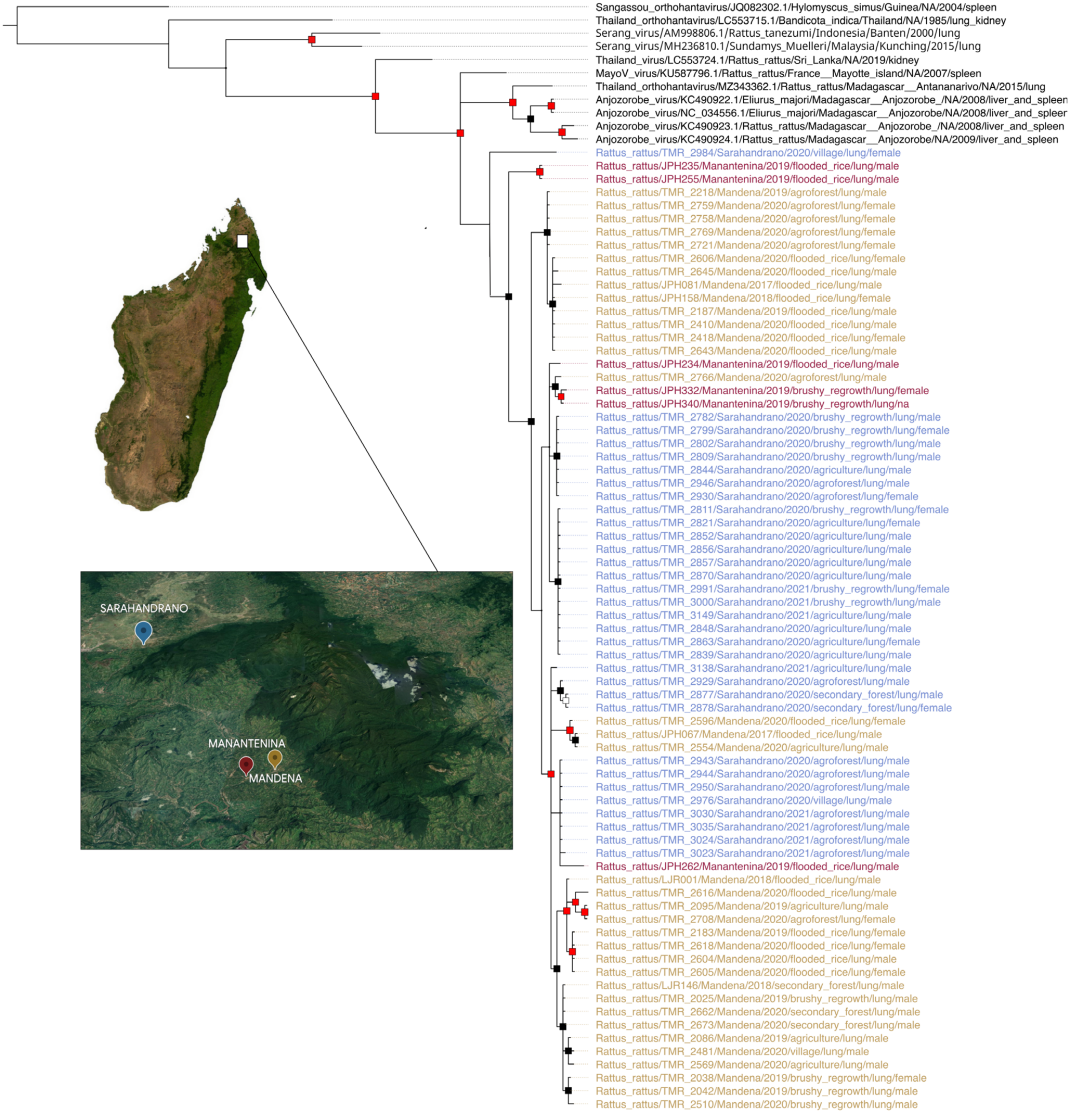
Bayesian phylogeny of hantavirus samples. Bayesian phylogeny showing relatedness of hantaviruses from within and near the Marojejy National Park area to ThaiV and its descendants. The outgroup (black) is a Sangassou Virus. The support level shows posterior probability. Nodes with a posterior probability > 0.75 are represented with black squares while red squares represent nodes with probabilities > 0.95. Specimens from Mandena are in yellow, Manantenina in red, and Sarahandrano in blue, and all are from *Rattus rattus*. Accession numbers are listed in Table S5 and the sequence alignment is provided in the Supporting Information. Satellite images were produced using Google SNES/Airbus Maxar Technologies Data SIO, NOAA, US Navy, NGA, GEBCO.

All amplicons matching the expected size were sequenced, resulting in partial L segments of 347 bp. Genetic identity with previously reported hantaviruses from Madagascar (Reynes et al. 2014; Raharinosy et al. 2018; Kikuchi et al. 2021) ranged between 85.0% and 91.6%. Identity restricted to our study cohort only (75 sequences) ranged from 89.6% to 100%. Forty‐one of the 75 obtained sequences were unique, indicating a high level of diversity. Our sequences did not form a phylogenetic cluster with hantaviruses known from nearby islands (Figure 1).

### 3.3 *Rattus rattus* Traits and Infections by Land‐Use Type

**FIGURE 2:**
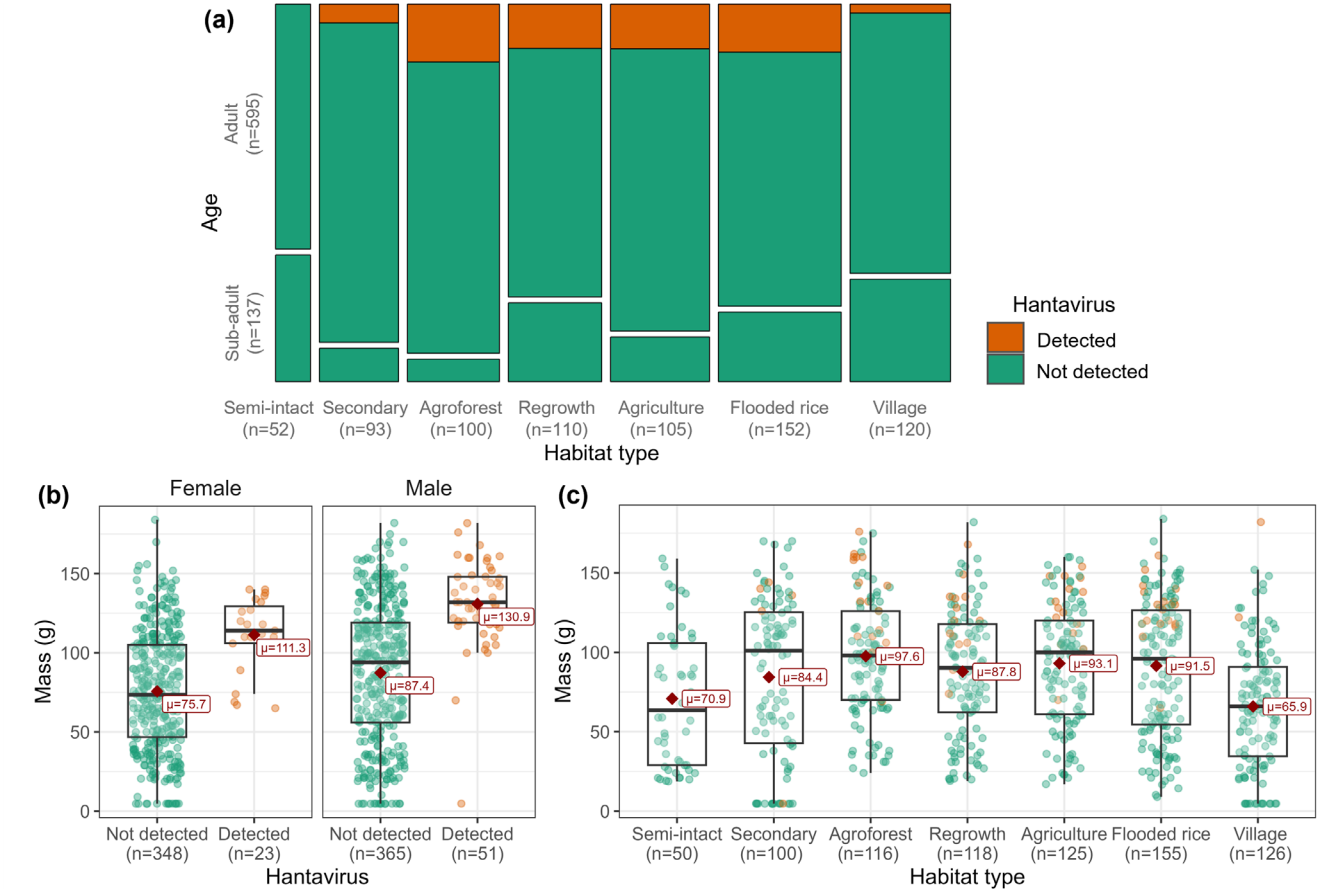
Hantavirus infection rates by (a) age class, (b) body mass and sex, and (c) body mass and habitat type. Orange represents animals in which hantavirus was detected and green those in which it was not detected. The average (μ) mass (g) is displayed on (b) and (c) and indicated with a red diamond. Individuals with missing age information (*n* = 62) were omitted from plot (a) and individuals with missing mass information (*n* = 4) were omitted from the plot (b) and (c).

*Rattus rattus* was the only species found to be infected with hantavirus; thus, we focused our analyses on this species. We detected hantavirus in 9.5% (75/794, 95% CI: 7.5%–11.7%) of *R. rattus* and infection rates varied with environmental and demographic factors, as well as animal density (Figure 2, Table S3). The age of rats varied significantly across land‐use type (Fisher's exact test, *p* < 0.001; Figure 2a) and we only detected hantavirus in adult rats. The average body mass of infected rats (125.0 g) was significantly greater than uninfected individuals (81.7 g; Figure 2b; Welch's two‐sample *t*‐tests, *p* < 0.001). The average mass of *R. rattus* varied significantly by habitat type (Figure 2c; Kruskal–Wallis, *p* < 0.001) and the infected individuals in each habitat type were mostly of above average mass. The same trends displayed in Figure 2b and Figure 2c were observed for head‐body length (mm) but not for body condition (g/mm^3^); see Figure S1 and Figure S2.

**FIGURE 3:**
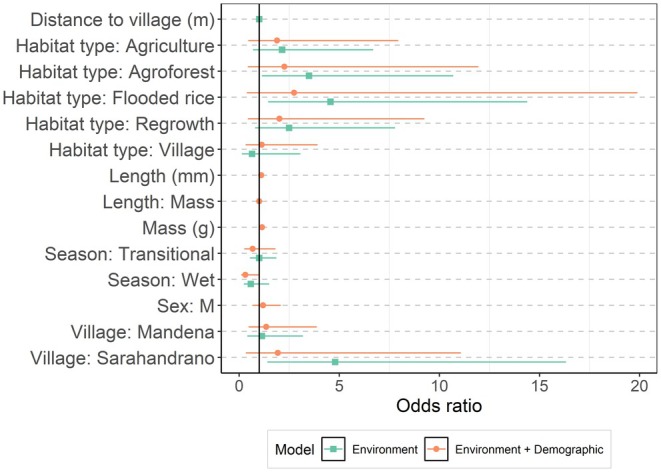
Estimated coefficients of the GLMMs. Full model‐averaged coefficients for the GLMM of models containing environmental (green squares) and environmental as well as demographic (orange circles) predictors. The coefficients (shapes) are shown with 95% confidence intervals. Figure S3 shows comparisons of all four models considered.

**FIGURE 4:**
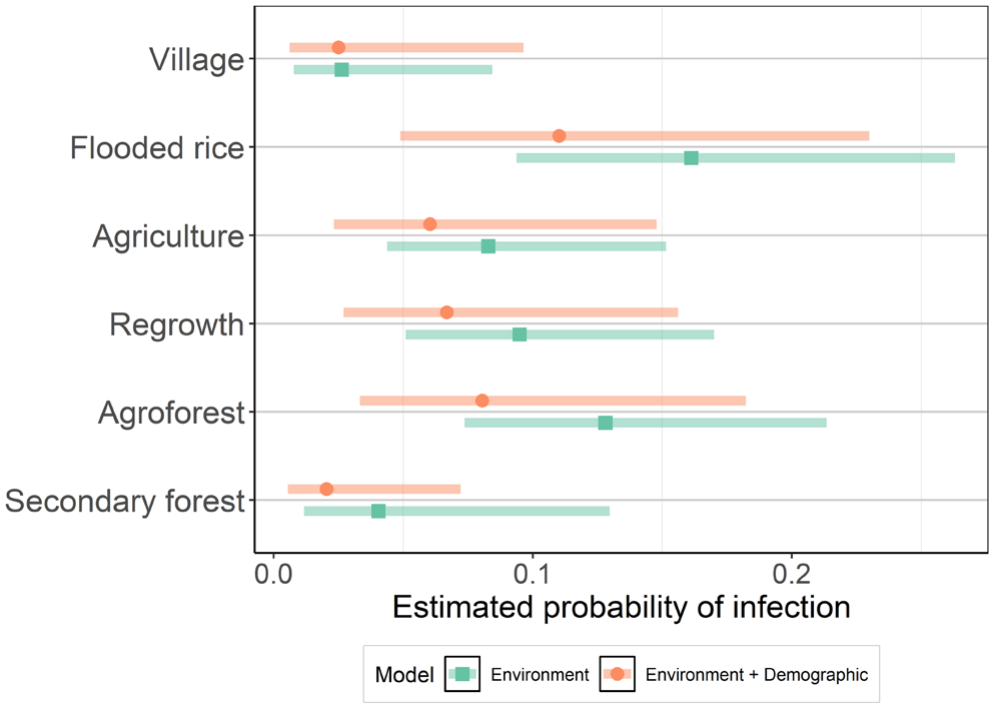
Predicted probability of Hantavirus infection in *Rattus rattus* across habitat types. Estimated marginal means probability of hantavirus infection across the different habitat types after accounting for variability in infection status due to other variables in the respective models, with a Tukey adjustment for multiple comparisons. The post hoc estimate made by the environment model (green square) and environment + demographic model (orange circle) and shaded areas of their corresponding 95% confidence intervals is displayed. Figure S4 shows comparisons of all four models considered and other categorical predictors.

The model of infection by the environmental traits (habitat type, season, village, and trap distance to village) showed that land use and village were important predictors of infection. The best‐models subset for the environmental covariates contained a model with habitat type, season, and village (AICc weight 0.501); a model with habitat type and village (AICc weight 0.310); and the global model with all variables (AICc weight 0.189). The most important predictors were habitat type and village, which were present in all models (sum weights 1.00). The season and distance to the village had respective weights of 0.69 and 0.19. The full model‐averaged estimates are shown in Figure 3 and Table 1. Post hoc comparisons between habitat types (Figure 4) revealed that the probability of infection was highest in flooded rice fields (0.16 ± 0.32) and agroforest (0.13 ± 0.31), and lowest in secondary forests (0.04 ± 0.64) and villages (0.03 ± 0.63). The infection level between flood rice fields and village habitat types was significantly different (*p* = 0.03). Semi‐intact forests were excluded from this analysis because there were no positive animals captured there. The probability of infection in Sarahandrano (0.18 ± 0.26) was significantly higher than in both Mandena (0.05 ± 0.26, *p* = 0.004) and Manantenina (0.04 ± 0.55, *p* = 0.04). While there was no significant post hoc pairwise difference in the probability of infection between the three seasons, the wet season tended to have lower infection odds (0.05 ± 0.41) than the dry (0.10 ± 0.30) and transitional seasons (0.10 ± 0.26; *p* = 0.10, 0.22 respectively).

**TABLE 1:** Importance and effect of variables in the environment, demographic, and environment + demographic models.

	Model		
	Environmental	Demographic	Environmental + demographic
Habitat type			
Secondary forest	(reference)		(reference)
Agroforest	3.49 [1.14, 10.69]		2.26 [0.43, 11.95]
Regrowth	2.49 [0.79, 7.79]		2.01 [0.44, 9.26]
Agriculture	2.15 [0.69, 6.7]		1.89 [0.45, 7.94]
Flooded rice	4.56 [1.44, 14.39]		2.75 [0.38, 19.89]
Village	0.64 [0.13, 3.06]		1.13 [0.32, 3.92]
Season			
Dry	(reference)		(reference)
Wet	0.58 [0.23, 1.49]		0.31 [0.1, 0.98]
Transitional	1 [0.54, 1.85]		0.68 [0.25, 1.81]
Village			
Manantenina	(reference)		(reference)
Mandena	1.14 [0.41, 3.18]		1.35 [0.47, 3.87]
Sarahandrano	4.79 [1.41, 16.33]		1.93 [0.34, 11.08]
Distance to village (m)			
	1 [1, 1]		
Number of rats			
Sex			
Female		(reference)	(reference)
Male		1.12 [0.7, 1.8]	1.19 [0.68, 2.08]
Mass (g)			
		1.13 [1.03, 1.24]	1.14 [1.04, 1.26]
Length (mm)			
		1.1 [1.02, 1.18]	1.11 [1.02, 1.19]
Length: Mass			
		1 [1, 1]	1 [1, 1]
Body condition (g/mm^3^)			
		1.01 [0.69, 1.49]	
Model fit			
AICc	451.9	387.66	384.59
Nakagawa's marginal R^2^	0.21	0.64	0.73

*Note:* Importance: 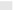 NA; 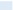 0–0.19; 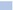
0.20–0.39; 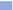 0.40–0.59; 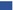 0.60–0.79; 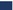 0.80–1.00.

The model of infection as predicted by morphometric traits (sex, body condition, mass, head‐body length, and the interaction between mass and length), explained a greater proportion of variance (Nakagawa's marginal *R* ^2^ = 0.64) than the environmental model (Nakagawa's marginal *R* ^2^ = 0.21; Table 1). The best model subset of the 12 possible models of individual‐level predictors of infection included a model with age, mass, and their interaction (AICc weight 0.435) and four other models with AICc weights < 0.3 (Figure S3 and Table 1). Mass and head‐body length were the most important predictors and were present in all models (cumulative sum weight = 1) followed by their interaction (cumulative sum weight = 0.97). Sex and body condition score were in two models, with sum weights of 0.37 and 0.26, respectively.

### 3.4 Individual and Environmental Predictors of Infection

The morphometric measurements of trapped *R. rattus* covaried with the environmental predictors of infection, namely habitat types, seasons, and villages. To address whether the observed differences in the probability of infection between habitat types were due to demographic variability, rat density, or a combination of these effects, three additional GLMM averaged results were considered. Of these three models, the best fit GLMM combined environmental (habitat types, seasons, and villages) and demographic (mass, head‐body length, their interaction, and sex) effects. The results from the other two models are shown in Figure S3 and Table S4.

Combining the important individual‐level and environmental predictors explained more of the variability in the dataset (Nakagawa's marginal *R* ^2^ = 0.73) than either the individual‐level predictors or the environmental level predictors alone (Table 1). The most important predictors in this combined model across the 16 models in the best‐models subset were head‐body length and mass (sum weights of 1), followed by their interaction term (14 models; sum weight = 0.98). The next important effects were season (10 models, sum weight = 0.90), habitat type (6 models, sum weight = 0.57), village (7 models, sum weight = 0.46), and sex (7 models, sum weight = 0.44). Post hoc comparisons of the probability of infection between habitat types show that the effect of habitat type is minimized when demographic effects are included in the model (Figure 4). The differences in infections between seasons were significant in this model, with the odds of infection in the dry season 3.68 ± 1.59 time greater than in the wet season (*p* = 0.01). Altogether the analysis suggests the strongest predictors of infection are morphometrics, with larger rats, regardless of sex, being more infected than small rats.

## 4 Discussion

Our extensive investigation of hantavirus in small mammals from northeastern Madagascar only detected infections in *Rattus rattus* (9.5%) despite testing over 20 species of terrestrial mammals, composed of 1663 individuals (including 450 endemics) and 227 native bats. The detected hantaviruses were distinct but genetically nested within the clade containing ANJZV, MayoV, and ThaiV sequences, which were previously described on Madagascar and Mayotte (Reynes et al. 2014; Filippone et al. 2016; Raharinosy et al. 2018; Kikuchi et al. 2021). The viral lineage found in *R. rattus* from our samples in the Marojejy National Park area in northeast Madagascar shows less than 92% identity with ANJZV from central eastern Madagascar, located 480 km southwest of the study site, and MayoV from Mayotte Island, located 530 km northwest of the study site. However, longer sequences are needed to robustly establish the genetic relationships of the virus occurring in the Marojejy area and the previously reported ANJZV and MayoV.

The absence of hantavirus detection in any of the endemic or native species may be due to extremely low prevalence or the presence of a distant viral lineage that could not be detected with our PCR scheme. We screened 269 of the 450 trapped endemic terrestrial mammals with three alternative semi‐nested PCR schemes, which still led to negative results (Table S1). ANJZV has been previously reported from the endemic rodent *Eliurus majori* (n tested = 15) in the Central Highlands of Madagascar with the same PCR scheme used herein (Reynes et al. 2014). Since hantaviruses are notorious for host switching thanks to their tri‐partite genome (Guo et al. 2013), we can hypothesize that the absence of detectable virus in endemic animals mirrors a recent introduction to Madagascar, where adaptation to the endemic species is in an early stage. However, further investigations are needed to date the introduction of the virus.

Hantavirus infection in our study was similar to other studies of the virus in Madagascar (Reynes et al. 2014; Raharinosy et al. 2018). We found an infection rate of 9.5% in *R. rattus* , which is not significantly different from a previous study reporting an infection in *R. rattus* of 12.4% across the island (*χ* ^2^ = 2.7, *p* = 0.10) and 5.8% in Sambava (*χ* ^2^ = 0.69, *p* = 0.41), in close proximity to our study site (Raharinosy et al. 2018). The *R. rattus* association we observed was also reported by Reynes et al. (2014), however, they detected Hantavirus in one endemic species ( *E. majori* ) which is found in higher elevation humid forests than where we trapped animals in this study. Though season does not significantly impact Hantavirus infections across Madagascar (Raharinosy et al. 2018), we found that infection rates tended to be significantly lower in the wet compared to the dry season once body size is accounted for, which suggests that variability in body size moderates the relationship between infection and season. *R. rattus* primarily reproduces during the wet season (Scobie et al. 2024) which suggests mating behaviors are not facilitating higher transmission rates as observed elsewhere (Jonsson, Figueiredo, and Vapalahti 2010).

Our sampling schema across a matrix of land‐use types allowed us to highlight determinants of infection at a finer scale than in previous studies (Reynes et al. 2014; Raharinosy et al. 2018). We found that agricultural land‐use types (agroforestry, brushy regrowth, agriculture, and flooded rice fields) contained a higher proportion of infected individuals than the most disturbed (village) and least disturbed (semi‐intact and secondary forest) habitats. This effect weakened, however, when the variability in animal body size between land‐use types was considered. The findings involving body size highlight the importance of demographic factors in reservoir populations for understanding prevalence and spillover risk, while also identifying potential mechanisms that relate land use change to zoonotic disease risk. Of note, not a single *R. rattus* captured in the semi‐intact forest tested positive. The low infection rate in the village is in accordance with previous findings from Madagascar (Raharinosy et al. 2018). In the Manantenina study area, statistically significant differences in body size of *R. rattus* , based on cranio‐dental measurements, have been identified between native forest and anthropogenic habitats (Ranaivoson et al. 2022). The niche breadth of animals living in natural forest was greater than in anthropogenic habitats (Dammhahn, Randriamoria, and Goodman 2017), presumably indicating a more stable diet during periods of seasonal variation and driving higher life expectancy. Hence, the longer‐lived animals have a greater chance of coming in contact with the virus, which is further substantiated by the absence of infection that we report in sub‐adult rats (*N* = 137, Figure 1). Alternatively, we cannot exclude that older males tend to leave the villages or are more at risk to be killed by domestic animals and people, hence contributing to lower overall infection in such rat populations.

Based on these findings, we propose that differences in hantavirus infection rates in *R. rattus* across our habitat gradient are due to a succession of synergetic biotic and abiotic factors, including resource availability, animal density, life expectancy, and level of habitat disturbance. Together these factors impacted population demography, which appears to drive infection in this system. Disturbances that alter the population demography to favor larger‐bodied and presumably older individuals may increase infection rates and thus human exposure risk. Based on the results that infection was highest in agricultural fields, we also expect that human exposure risk is highest when conducting farm‐based activities that aerosolize excreta or bring people into contact with *R. rattus*.

## 5 Conclusions

*Rattus rattus* is a remarkably adaptable species and appears to be the principal reservoir of hantavirus in the area surrounding Marojejy National Park in northeastern Madagascar. The positive individuals from our study formed a subclade of MayoV and ANJZV. Hantavirus infection in *R. rattus* varied across the land‐use matrix and was higher in agricultural areas than in forests and villages. Likewise, agricultural land‐use types had higher abundances of *R. rattus* than forested areas. However, the larger body size of *R. rattus* living in agricultural land‐use types compared to forests and villages likely explained the increased viral load. Differences in body size likely indicate a longer lifespan and increased odds of viral exposure. As invasive rat populations and interactions between endemic and introduced species continue to grow due to the ongoing conversion of forests into agricultural land, we expect hantavirus exposure risk to humans to increase, particularly when these changes positively alter *R. rattus* demography.

## Author Contributions

**Jérémy Dubrulle:** data curation (equal), formal analysis (equal), methodology (equal), writing – original draft (equal), writing – review and editing (equal). **Kayla Kauffman:** conceptualization (equal), data curation (equal), formal analysis (equal), investigation (equal), validation (equal), visualization (equal), writing – original draft (equal), writing – review and editing (equal). **Voahangy Soarimalala:** conceptualization (equal), funding acquisition (equal), investigation (equal), methodology (equal), writing – original draft (equal), writing – review and editing (equal). **Toky Randriamoria:** investigation (equal), methodology (equal), writing – review and editing (equal). **Steven M. Goodman:** conceptualization (equal), data curation (equal), funding acquisition (equal), investigation (equal), methodology (equal), writing – original draft (equal), writing – review and editing (equal). **James Herrera:** data curation (equal), formal analysis (equal), investigation (equal), methodology (equal), writing – original draft (equal), writing – review and editing (equal). **Charles Nunn:** conceptualization (equal), formal analysis (equal), funding acquisition (equal), investigation (equal), methodology (equal), project administration (equal), supervision (equal), validation (equal), writing – original draft (equal), writing – review and editing (equal). **Pablo Tortosa:** conceptualization (equal), formal analysis (equal), funding acquisition (equal), methodology (equal), supervision (equal), validation (equal), writing – original draft (equal), writing – review and editing (equal).

## Conflicts of Interest

The authors declare no conflicts of interest.

## Supporting information


Figure S1.

Figure S2.

Figure S3.

Figure S4.

Table S1.

Table S2.

Table S3.

Table S4.

Table S5.

## Data Availability

All genomic information in this manuscript is available on NCBI.
