# Supplementary figures and images for: Effect of Land‐Use on Hantavirus Infection Among Introduced and Endemic Small Mammals of Madagascar
Source: Ecol Evol. 2025 Apr 7;15(4):e70914. doi: 10.1002/ece3.70914 (PMC11975053; doi:10.1002/ece3.70914)

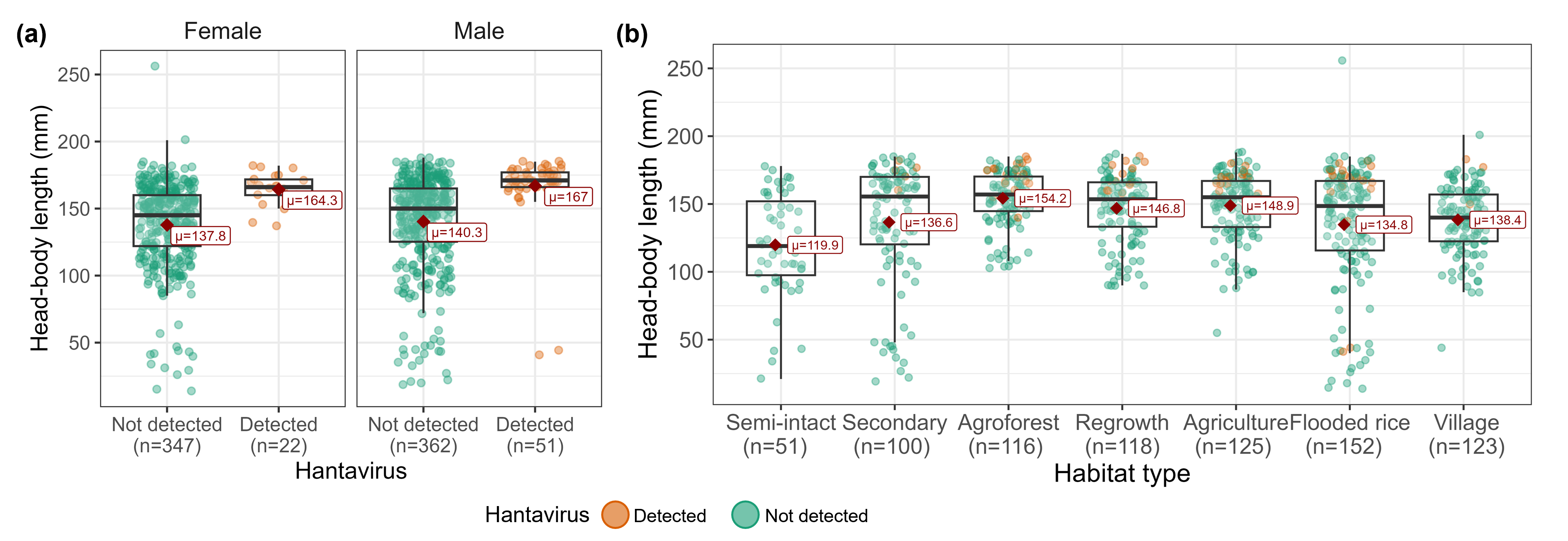

Supplement: Supplementary file 1 — Figure S1. [file ECE3-15-e70914-s007.tif]

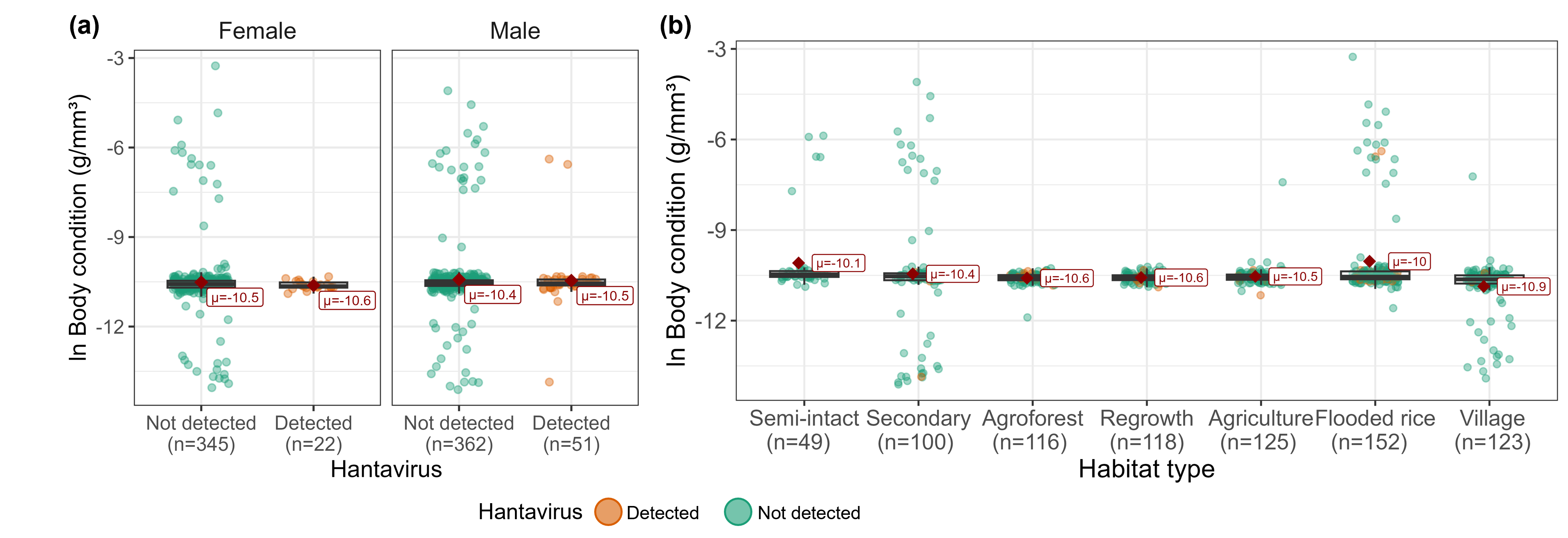

Supplement: Supplementary file 2 — Figure S2. [file ECE3-15-e70914-s005.tif]

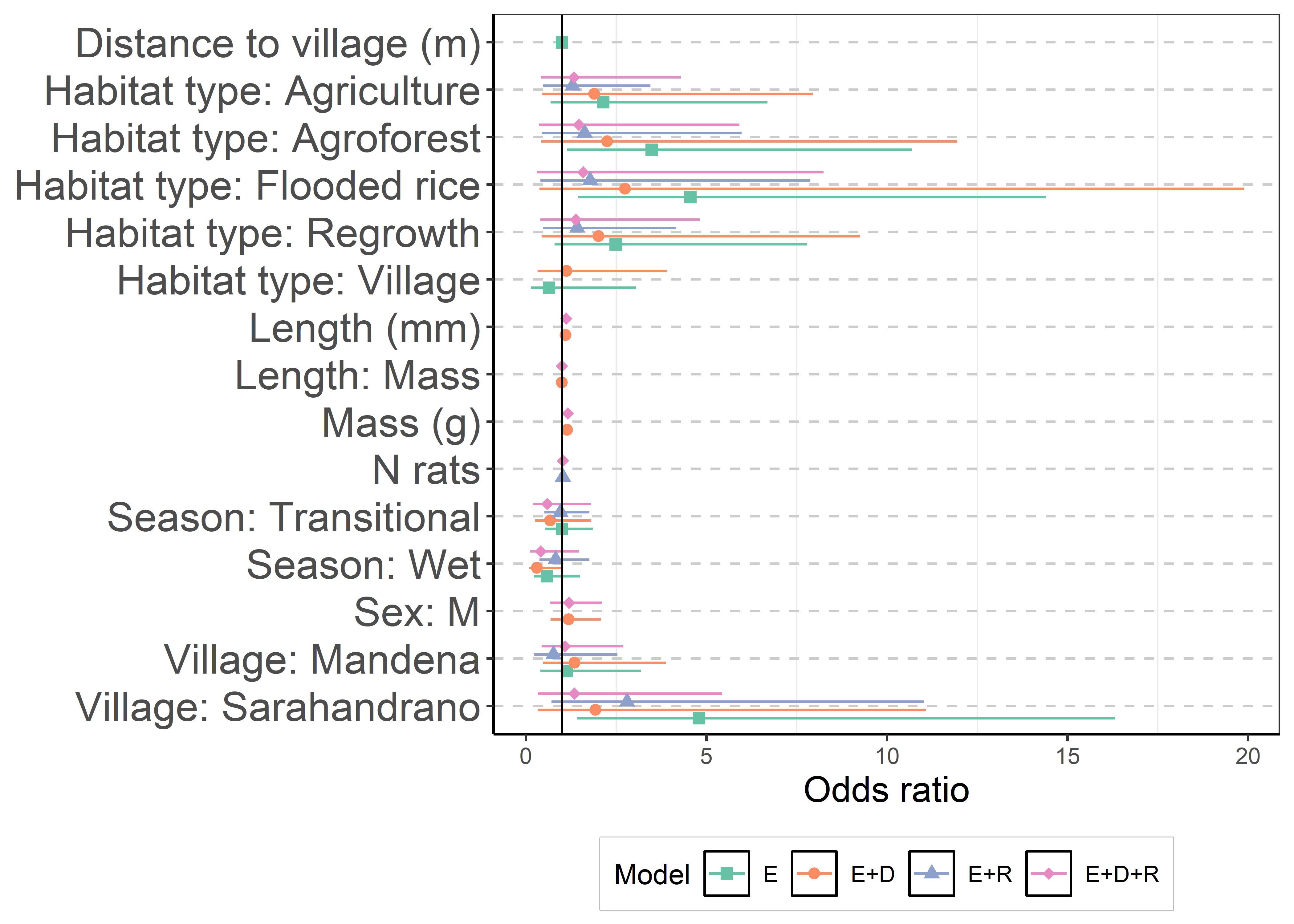

Supplement: Supplementary file 3 — Figure S3. [file ECE3-15-e70914-s001.tiff]

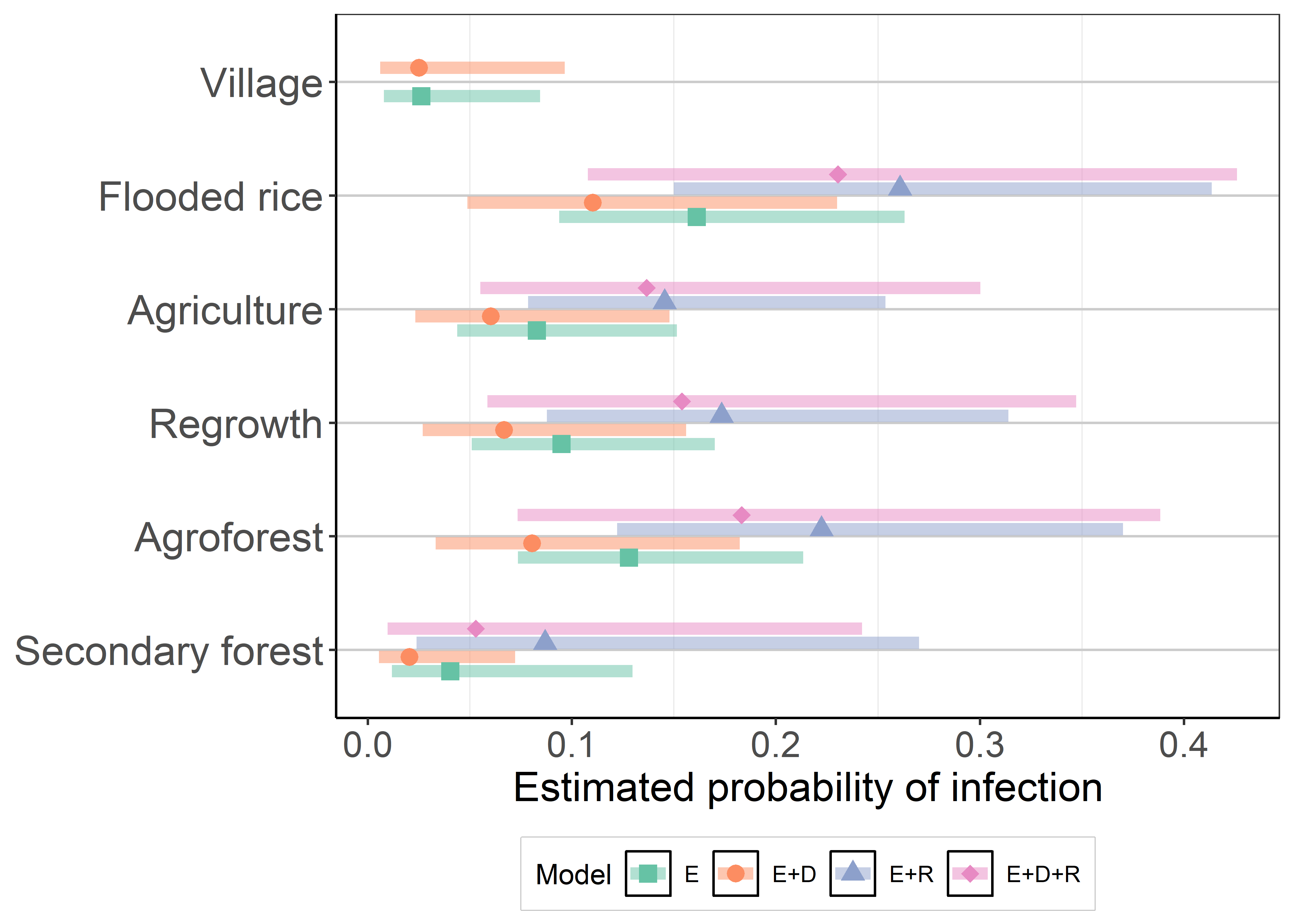

Supplement: Supplementary file 4 — Figure S4. [file ECE3-15-e70914-s004.tiff]
